# Abdominal Functional Electrical Stimulation to Assist Ventilator Weaning in Acute Tetraplegia: A Cohort Study

**DOI:** 10.1371/journal.pone.0128589

**Published:** 2015-06-05

**Authors:** Euan J. McCaughey, Helen R. Berry, Alan N. McLean, David B. Allan, Henrik Gollee

**Affiliations:** 1 Centre for Rehabilitation Engineering, School of Engineering, University of Glasgow, University Avenue, Glasgow, United Kingdom; 2 Centre for Excellence in Rehabilitation Research, Biomedical Engineering, University of Strathclyde, Glasgow, United Kingdom; 3 Centre for Health Systems and Safety Research, Australia Institute of Health Innovation, Macquarie University, North Ryde, Australia; 4 Queen Elizabeth National Spinal Injuries Unit, Southern General Hospital, Glasgow, United Kingdom; 5 Scottish Centre for Innovation in Spinal Cord Injury, Glasgow, United Kingdom; Erasmus Medical Centre, NETHERLANDS

## Abstract

**Background:**

Severe impairment of the major respiratory muscles resulting from tetraplegia reduces respiratory function, causing many people with tetraplegia to require mechanical ventilation during the acute stage of injury. Abdominal Functional Electrical Stimulation (AFES) can improve respiratory function in non-ventilated patients with sub-acute and chronic tetraplegia. The aim of this study was to investigate the clinical feasibility of using an AFES training program to improve respiratory function and assist ventilator weaning in acute tetraplegia.

**Methods:**

AFES was applied for between 20 and 40 minutes per day, five times per week on four alternate weeks, with 10 acute ventilator dependent tetraplegic participants. Each participant was matched retrospectively with a ventilator dependent tetraplegic control, based on injury level, age and sex. Tidal Volume (V*_T_*) and Vital Capacity (V*_C_*) were measured weekly, with weaning progress compared to the controls.

**Results:**

Compliance to training sessions was 96.7%. Stimulated V*_T_* was significantly greater than unstimulated V*_T_*. V*_T_* and V*_C_* increased throughout the study, with mean V*_C_* increasing significantly (V*_T_*: 6.2 mL/kg to 7.8 mL/kg V*_C_*: 12.6 mL/kg to 18.7 mL/kg). Intervention participants weaned from mechanical ventilation on average 11 (sd: ± 23) days faster than their matched controls.

**Conclusion:**

The results of this study indicate that AFES is a clinically feasible technique for acute ventilator dependent tetraplegic patients and that this intervention may improve respiratory function and enable faster weaning from mechanical ventilation.

**Trial Registration:**

ClinicalTrials.gov NCT02200393

## Introduction

An injury to the cervical region of the spinal cord can cause paralysis affecting all four limbs and the trunk, termed tetraplegia. People with tetraplegia have paralysis and impaired function of the major respiratory muscles, namely the diaphragm and the intercostal and abdominal muscles. This reduces respiratory function, resulting in up to 40% of these individuals requiring mechanical ventilation in the acute stage of injury [1]. While approximately 75% of these patients will wean from mechanical ventilation, its use is associated with increased likelihood of respiratory infection and a three and a half fold increase in mortality rate compared to the general spinal cord injured population [2–4]. Ventilator dependence also reduces quality of life, delays rehabilitation and increases costs for the local health care provider (by approximately $1500 per day) [5,6]. Any improvement in respiratory function that reduces the time dependent on mechanical ventilation will have numerous benefits.

Functional Electrical Stimulation (FES) is the application of a train of electrical pulses to a motor nerve, causing the associated muscle to contract [7]. Routsi et al. [8] demonstrated that the application of FES to muscles in the legs of 24 critically ill ventilator dependent patients resulted in these patients weaning from mechanical ventilation at a statistically significantly faster rate than matched controls. The application of FES to the abdominal muscles is called Abdominal FES (AFES). In a number of pilot studies, the repeated application of AFES, termed an AFES training program, has been shown to improve the respiratory function of different tetraplegic patient groups. McBain *et al*. [9] demonstrated that an AFES training program increased the esophageal and gastric expiratory pressures and the Peak Expiratory Flow (PEF) generated by non-ventilator dependent participants with chronic (greater than three months post injury [10]) tetraplegia. An AFES training program has also been used to improve the Forced Vital Capacity (FVC) and PEF of patients with sub-acute (between four weeks and three months post injury) tetraplegia [11]. Lee et al. [12] demonstrated that an AFES training program could be used to assist ventilator weaning in one patient with chronic tetraplegia.

For tetraplegic patients who retain diaphragm function but require mechanical ventilation in the acute stage of injury due to respiratory failure, the duration of mechanical ventilation is variable. In a study of 73 such patients, where no intervention was applied, the mean ventilation duration was 37 days [13]. Spontaneous Breathing Trials (SBTs), combined with traditional airway physiotherapy, are commonly used to promote weaning from mechanical ventilation and reduce ventilation duration [14]. We propose that the combination of an AFES training program and SBTs will increase movement of the diaphragm, thereby improving lung function for the acute (less than four weeks post injury [15]) ventilator dependent tetraplegic population. This should lead to an increase in Tidal Volume (V_*T*_ *)* and V_*C*_ and reduce ventilation duration. Comparison of ventilation duration with matched controls should indicate the effectiveness of this intervention in assisting ventilator weaning.

The aim of this study was to evaluate the feasibility and effectiveness of an AFES training program to improve respiratory function and assist ventilator weaning in acute tetraplegia.

## Methods

### Participants

Ten tetraplegic patients, who were acute inpatients in the high dependency ward of a dedicated spinal injuries unit within a university teaching hospital were recruited between January 2012 and November 2013. All patients were ventilator dependent for a period of at least 24 hours, had no spontaneous breathing activity and had no useful abdominal movement. This spinal injuries unit is set to administer acute care and rehabilitation for all spinal injuries occurring nationally, with 47% of patients being admitted within 48 hours of injury [16]. Due to the high levels of sedation administered to each participant their ‘Welfare Guardian’, as defined by the ethics committee, provided written informed consent, with consent later sought from the participant if they regained capacity within the study duration.

### Study design

**Fig 1:**
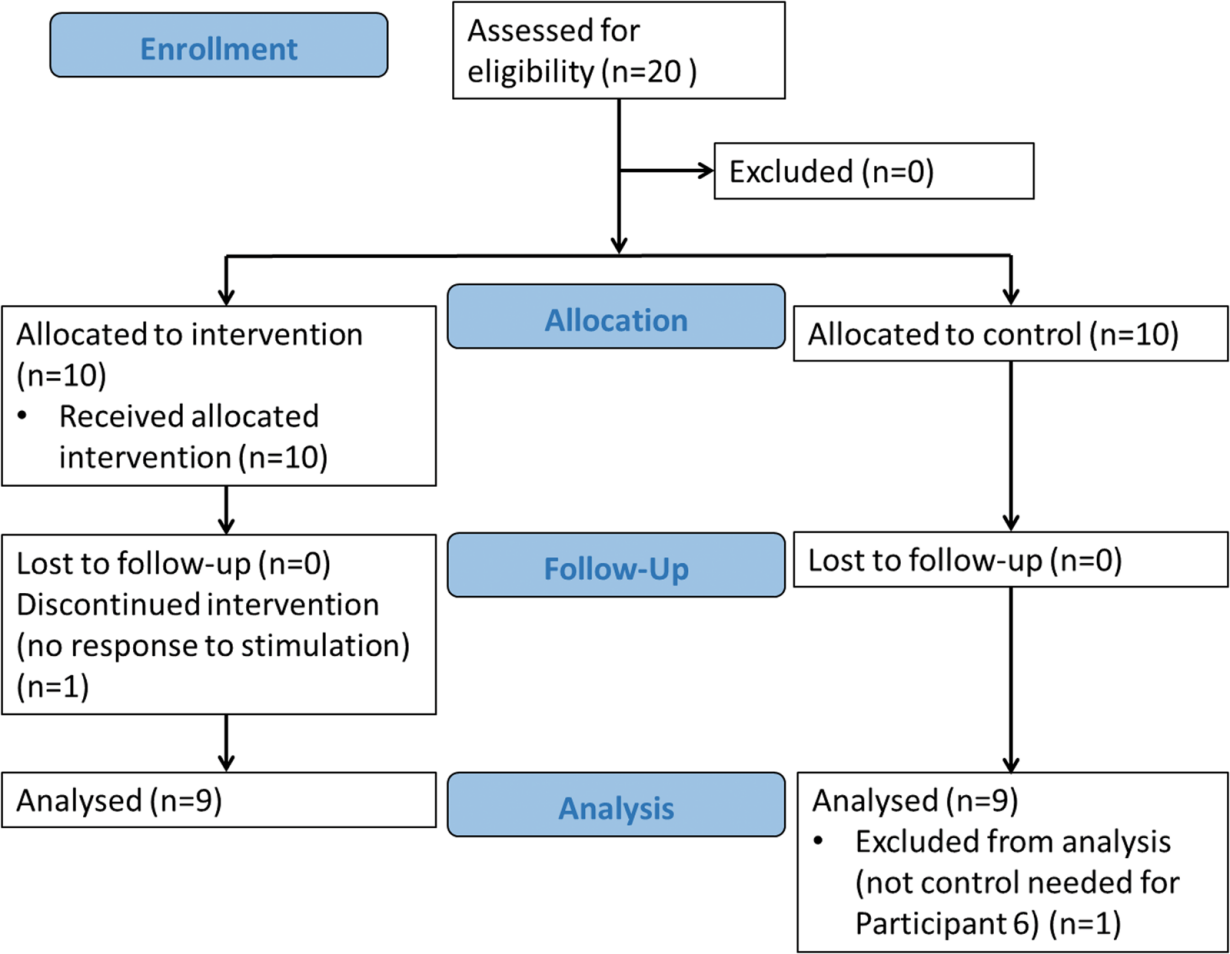
CONSORT flow diagram.

To enable a comparison of time to wean each participant was matched with a retrospective control who required mechanical ventilation in the acute stage of injury. Matching was determined by a spinal cord injuries consultant from information obtained from records of previous patients at the spinal unit. Matching was based on injury level, age (within five years) and sex; currently the best predictors of ventilation duration after spinal cord injury [17–19]. Due to the relatively small number of retrospective controls available within the spinal injuries unit, exact matches were difficult to achieve and matching was partially based on the clinical judgement of the consultant. The demographics of the intervention participants and their controls are shown in Table 1. Participant 5 did not complete the study due to being a non-responder to electrical stimulation (see below). A CONSORT flow diagram of this process is shown in Fig 1.

**Table 1:** Intervention and control demographics.

Intervention								Control			
Participant	Sex	Age (years)	Injury level	AIS level	Weight (kg)	Time post injury (days)	Time post ventilation (days)	Sex	Age (years)	Injury level	AIS level
1	F	42	C4	A	50	20	6	M	34	C5	A
2	M	63	C3/4	A	70	39	11	M	63	C3	C
3	M	38	C4	C	78	24	8	M	35	C5	A
4	M	53	C4	B	81	29	29	F	58	C4	A
5	M	44	C0/5	A	90	24	20	-	-	-	-
6	M	77	C3/4	C	76	10	9	M	78	C4	A
7	M	74	C6/7	C	82	43	40	M	73	C6	A
8	M	24	C5/6	A	80	11	7	M	25	C5	B
9	M	32	C5	B	95	11	8	M	32	C5	A
10	F	35	C7	A	75	11	2	M	32	C5	A
Mean±s.d.		48.2±18.0			77.7±12.1	22.2±12.0	14±12.0		47.8±20.2		

Time post injury and time post ventilation refers to time at first assessment session. Note: no control participant was sought for intervention Participant 5, as this participant did not complete the training protocol.

Abbreviations: AIS, American Spinal Injuries Association Impairment Scale (AIS score refers to function and sensation below injury level: A—no motor function or sensation, B—no motor function with sensation, C—severely compromised motor function with sensation.); S.d, standard deviation.

The study was approved by the NHS Scotland A Research Ethics Committee in January 2012. All experimental procedures conformed to the Declaration of Helsinki. The trial was registered retrospectively on ClinicalTrials.gov (NCT02200393, http://clinicaltrials.gov/ct2/show/NCT02200393), as facilities were not in place for registration at trial commencement. The authors confirm that all ongoing and related trials for this intervention are registered. The protocol for this trial and supporting TREND checklist are available as supporting information; see S1 Protocol and S1 Statement.

### Study protocol

**Fig 2:**
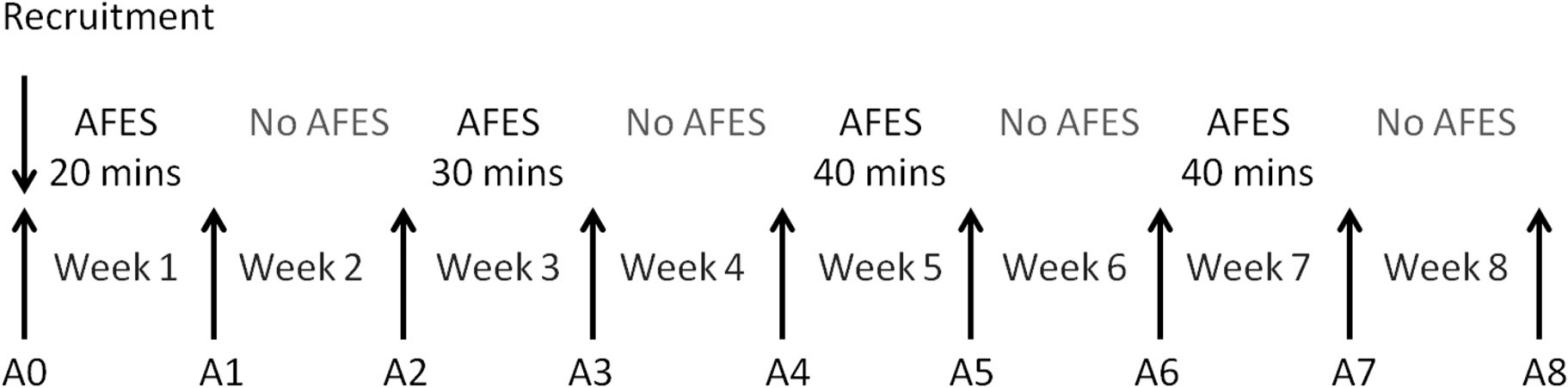
Study time line.

Each participant took part in an eight week program, involving AFES training sessions five times per week, on four alternate weeks (see Fig 2). Their respiratory function was measured at weekly assessment sessions. All procedures were performed at the participant’s bedside with the participant in a supine position (due to patients being mechanically ventilated and heavily sedated in early stages of study), and effort made to perform all procedures at the same time of day.

#### Training session

Due to the anticipated number of patients who would be eligible for this study within the study time frame it was not possible to have a dedicated control cohort. Therefore, AFES training was applied on alternate weeks, with patients serving as their own control. AFES training sessions were performed for 20 minutes per day in week one, 30 minutes per day in week three and 40 minutes per day in weeks five and seven (see Fig 2). It was anticipated that by comparing changes in respiratory function during weeks where AFES training was applied, with weeks where it was not applied, the effectiveness of the intervention could be estimated. During all training sessions stimulation was applied automatically at the start of exhalation (see Section Equipment for further details).

When participants were not able to breathe independently of mechanical ventilation they remained connected to mechanical ventilation throughout the training session. As SBTs began training sessions commenced with participants disconnected from mechanical ventilation. Throughout all training sessions the participant’s oxygen saturation level (SaPO_2_) was recorded every minute. If SaPO_2_ decreased below 92% participants were immediately reconnected to mechanical ventilation until SaPO_2_ returned to baseline, at which point they were once again disconnected from mechanical ventilation. AFES was applied throughout this process. Training sessions continued until completion of the eight week protocol, even if the participant weaned from mechanical ventilation during the study.

#### Assessment session

At the start of the study, and at the end of each week, an assessment session was conducted to measure the participant’s respiratory function. During each assessment session V_*T*_ and V_*C*_ were measured with and without AFES. Stimulated and unstimulated V_*T*_ were both recorded by asking participants to breathe normally for six minutes. These periods were separated by a break of two minutes, or until the participant’s SaPO_2_ returned to baseline, with participants ventilated during this break if required. Stimulated and unstimulated V_*T*_ were calculated as the mean of the values recorded during the respective assessment periods. When high levels of sedation rendered participants unable to actively participate, V_*T*_ and V_*C*_ were recorded from the ventilator. This occurred at the first two assessments with Participant 1, and the first assessment with Participant 6, with V_*T*_ calculated as the mean of six breaths recorded from the ventilator and V_*C*_ the maximum of these breaths.

To record both stimulated and unstimulated V_*C*_ participants were asked to inhale to maximum lung capacity and exhale fully. This was repeated until a minimum of three and a maximum of five successful manoeuvres (based on the guidelines of the American Thoracic Society/European Respiratory Society standards for spirometry [20]) were achieved. V_*C*_ was measured before and after the V_*T*_ procedures, resulting in a total of six to 10 V_*C*_ attempts for both stimulated and unstimulated breaths. Stimulated and unstimulated V_*C*_ were calculated as the maximum of the six to 10 successful attempts that were within 0.15 L of another attempt. V_*C*_ was recorded rather than FVC as participants could not always exhale as ‘forcibly as possible’ due to sedation levels and poor respiratory muscle coordination. However, there is little difference between these measures [21]. Whether stimulation was applied before or after recording non-stimulated measures was randomised for each participant at each assessment session.

### Time to wean

The total time each day that a participant spent breathing without the support of mechanical ventilation was recorded throughout the study and extracted from the medical notes of each control. Controls were followed up for 100 days post injury and deemed not to have weaned from mechanical ventilation if they were unable to breathe independently by this point. Weaning was defined as seven consecutive days without ventilator support [17].

### Equipment

**Fig 3:**
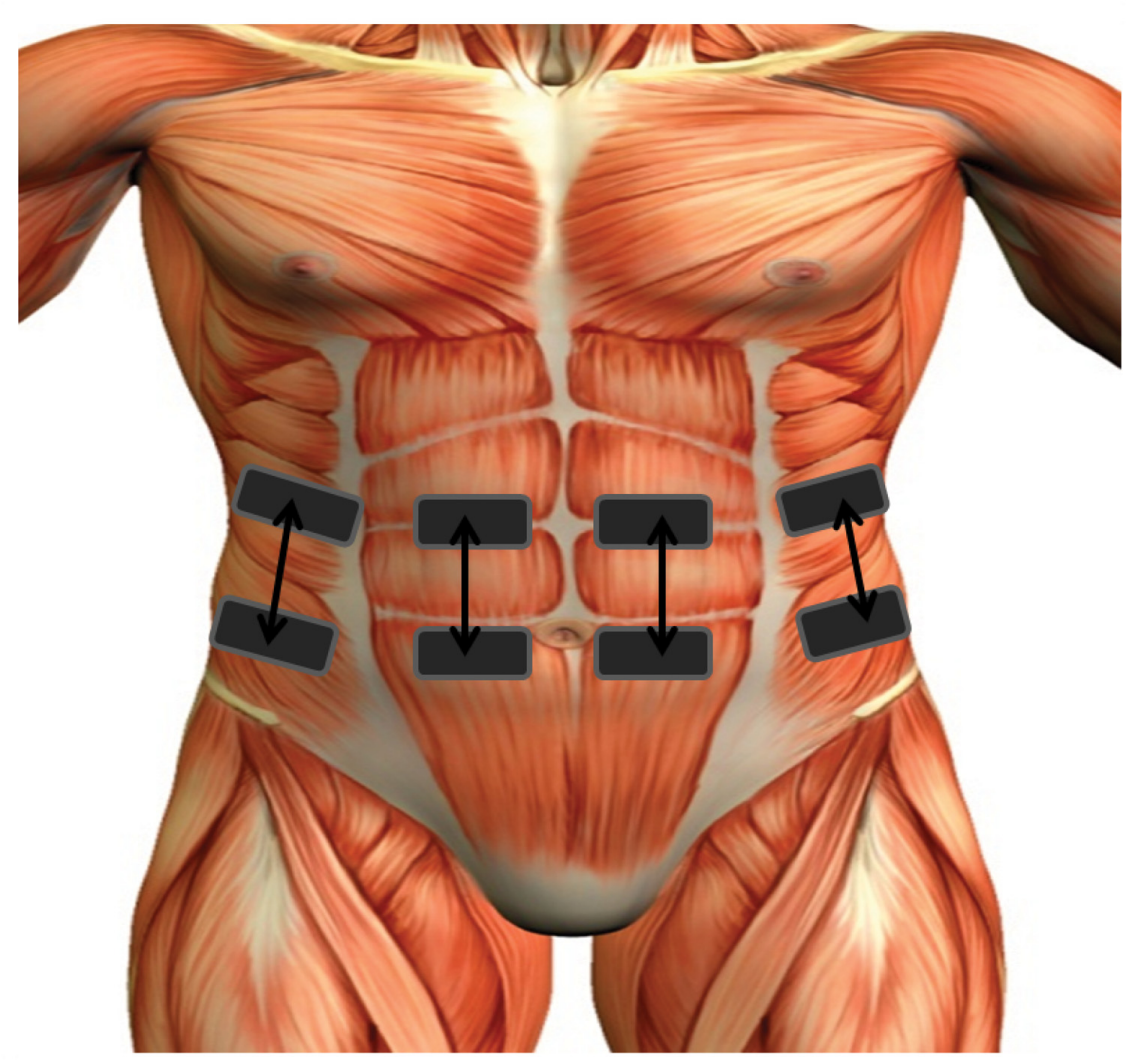
Schematic diagram of electrode placement.

A programmable neuromuscular stimulator (Rehastim v1, Hasomed, Germany) was used to stimulate the rectus abdominis and external oblique muscles bilaterally using four stimulation channels. Stimulation was applied during exhalation via surface electrodes (33 *mm* x 53 *mm* rectangular, PALS, Axelgaard, USA) placed over the motor points of the rectus abdominis and external oblique muscles on both sides of the body, as shown in Fig 3. Motor points were detected using a previously described technique [22].

**Fig 4:**
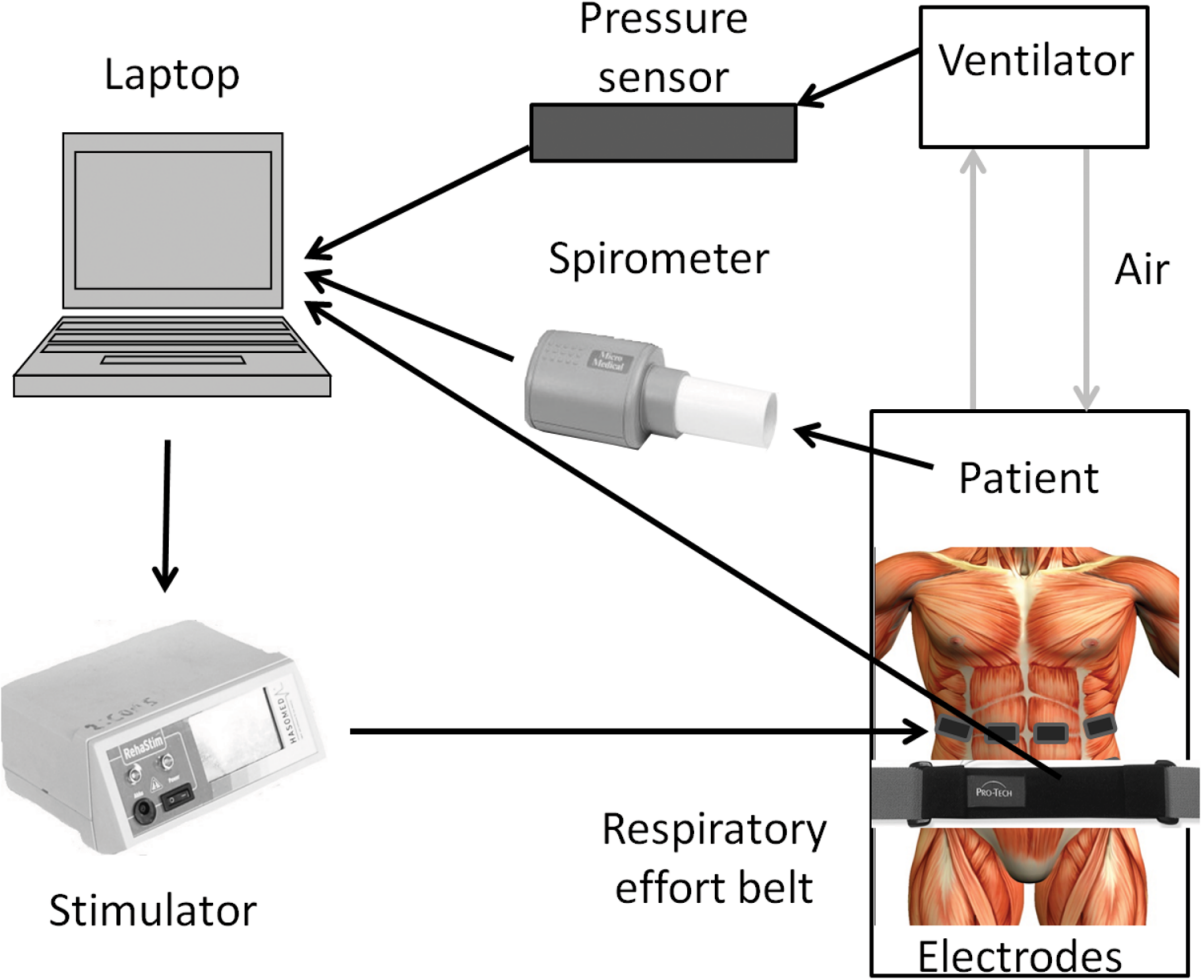
Sensors used to record respiratory activity. Black lines indicate direction of data.

While participants were dependent on mechanical ventilation the activity of the ventilator, representing the participant’s respiratory activity, was detected using a pressure sensor (HDIM050GBZ8H5, First Sensor AG, Germany) connected in line with the expiratory limb of the ventilator. When participants were breathing independently, respiratory activity was detected using a respiratory effort belt positioned around the abdomen (Piezoelectric belts, ProTech, USA) [23]. During assessment sessions respiratory function was measured using a spirometer (Microloop, Micromedical, UK). The methods used to detect respiratory activity are summarised in Fig 4. At initial assessment sessions all participants had a tracheostomy tube, to which the spirometer was connected. If the tracheostomy cuff was deflated, or a cuffless tracheostomy inserted, the spirometer was connected to a mouthpiece, with the tracheostomy tube capped to avoid air leakage. A mouthpiece was used to measure respiratory function after the tracheostomy tube was removed. This occurred within one week of weaning from mechanical ventilation for all participants.

Based on the detected respiratory activity, AFES was automatically applied at the start of exhalation for a duration of one second. Bi-phasic current controlled stimulation pulses were applied at a frequency of 30 Hz. Current was adjusted on a channel by channel basis (range 30–105 mA for all participants) with a pulsewidth of 100 *μ*s until a strong visible contraction of the abdominal muscles was observed. Pulsewidth was adjusted throughout each training session to account for muscle fatigue (100 − 500 *μ*s). Therefore, blinding was not relevant or practical for this intervention.

### Analysis and outcome measures

The primary outcome measure of this study was respiratory function (with and without stimulation). The secondary outcome measure was time to wean from mechanical ventilation. Respiratory function was quantified by V_*T*_ and V_*C*_, measured in litres (L). To allow group comparison these measures were normalised by weight and are presented as weight corrected tidal volume (V_*T*_/kg) and weight corrected vital capacity (V_*C*_/kg) [17–19], measured in millilitres per kilogram (mL/kg).

Group V_*T*_/kg and V_*C*_/kg data were tested for normality using a Ryan-Joiner test. Based on the results V_*C*_/kg data was transformed using a Johnson Transformation. A General Linear Mixed Model (GLMM) was used to test for significant differences between stimulated and unstimulated V_*T*_/kg and V_*C*_/kg and for longitudinal significant changes in V_*T*_/kg and V_*C*_/kg when controlling for assessment sessions and random variation between participants (model: V_*T*_/kg or V_*C*_/kg = AFES (factor) + Assessment (factor) + Subject (random factor) + error). In the case of longitudinal significance, post hoc multiple comparisons were performed using the Tukey-Kramer honest significant difference test to identify significantly different groups. Differences between stimulated and unstimulated responses at each assessment session were determined using a pairwise comparison, with a Tukey’s range test used to control for multiple comparisons.

A Cox proportional hazard model was developed using time to wean and group (intervention or no intervention). This model was used to compare the time for the intervention participants and their controls to achieve seven days of ventilator free breathing. Participants who did not wean from mechanical ventilation within the study duration (100 days) were censored.

For all tests a p-value of <0.05 was considered significant. Statistical testing was performed using Minitab (version 17.0, Minitab, State College, PA, USA) and SPSS (version 22.0, IBM, NY, USA) software.

The variability of time to wean from ventilation in the tetraplegic population and the fact that the quantitative effectiveness of the intervention was largely unknown in this patient group made it difficult to reliably estimate the required sample size. The target number of patients was therefore determined by an estimate of the available patient population at the spinal unit who would be eligible for this study.

## Results

Participant 5 did not respond to AFES due to lower motor neuron damage (detected through magnetic resonance imaging) as a result of ascending oedema of the spinal cord. No data was collected from this participant. Participant 1 developed pneumonia during week four of participation, missing one week of the study. The study was extended to nine weeks to account for this. Participant 7 did not complete the final assessment session due to an acute gastrointestinal haemorrhage. The results recorded during the previous assessment session were used for group analysis. In total 80 individual assessment sessions were recorded. The overall compliance rate to training sessions was 96.7%.

V_*T*_/kg was found to have a normal distribution (*p* > 0.1 for all tests), while V_*C*_/kg (*p* < 0.01) was found to significantly deviate from a normal distribution. As such, V_*C*_/kg was transformed using a Johnson Transformation (standard bounded distribution, *p* = 0.51).

### Tidal volume

**Fig 5:**
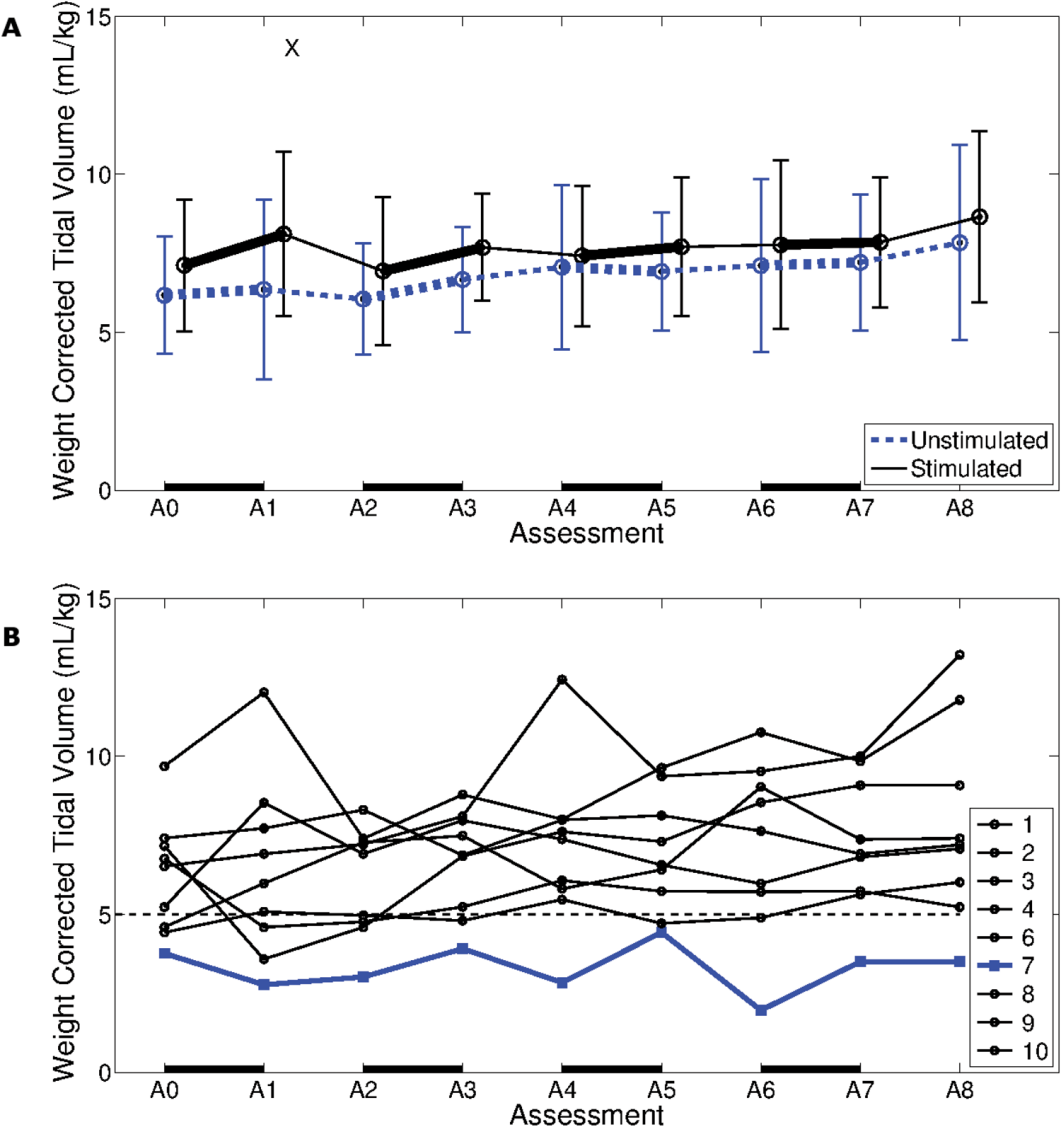
Weight corrected tidal volume. A. Group weight corrected tidal volume (VT /kg) (mean ± standard deviation) for 9 participants, recorded at 9 weekly assessment sessions. A black line represents stimulated, and a blue line unstimulated, breaths. A black X indicates stimulated VT /kg was statistically significantly different to unstimulated VT /kg. B. Unstimulated VT /kg of nine participants, recorded at nine weekly assessment sessions. Participant 7, who was the only participant not to wean from ventilation within the study duration, is represented by a blue line. A VT /kg of 5 mL/kg is represented by a dashed black line, with this value possibly serving as an indicator of weaning success. A solid black line along the bottom of both plots represents one week of AFES training.

Stimulated V_*T*_/kg was statistically significantly different to unstimulated V_*T*_/kg (p < 0.00). Stimulated V_*T*_/kg was significantly greater than unstimulated V_*T*_/kg at one of the nine assessment sessions (see Fig 5A). When controlling for assessment sessions (*p* < 0.01), and random variation between participants (*p* < 0.00), longitudinal changes in stimulated or unstimulated V_*T*_/kg (*p* = 0.07 and *p* = 0.09) were not significant. Stimulated V_*T*_/kg increased in all weeks where AFES training was applied, but only increased in the final two weeks when AFES training was not applied. The differences in unstimulated V_*T*_/kg was similar during weeks with and without stimulation. None of these differences were significant.

Participant 7, who had the lowest unstimulated V_*T*_/kg, with a mean of around 3 mL/kg across the study duration (see Fig 5B), was the only intervention participant who completed the training protocol and did not wean from mechanical ventilation within the study duration.

### Vital capacity

**Fig 6:**
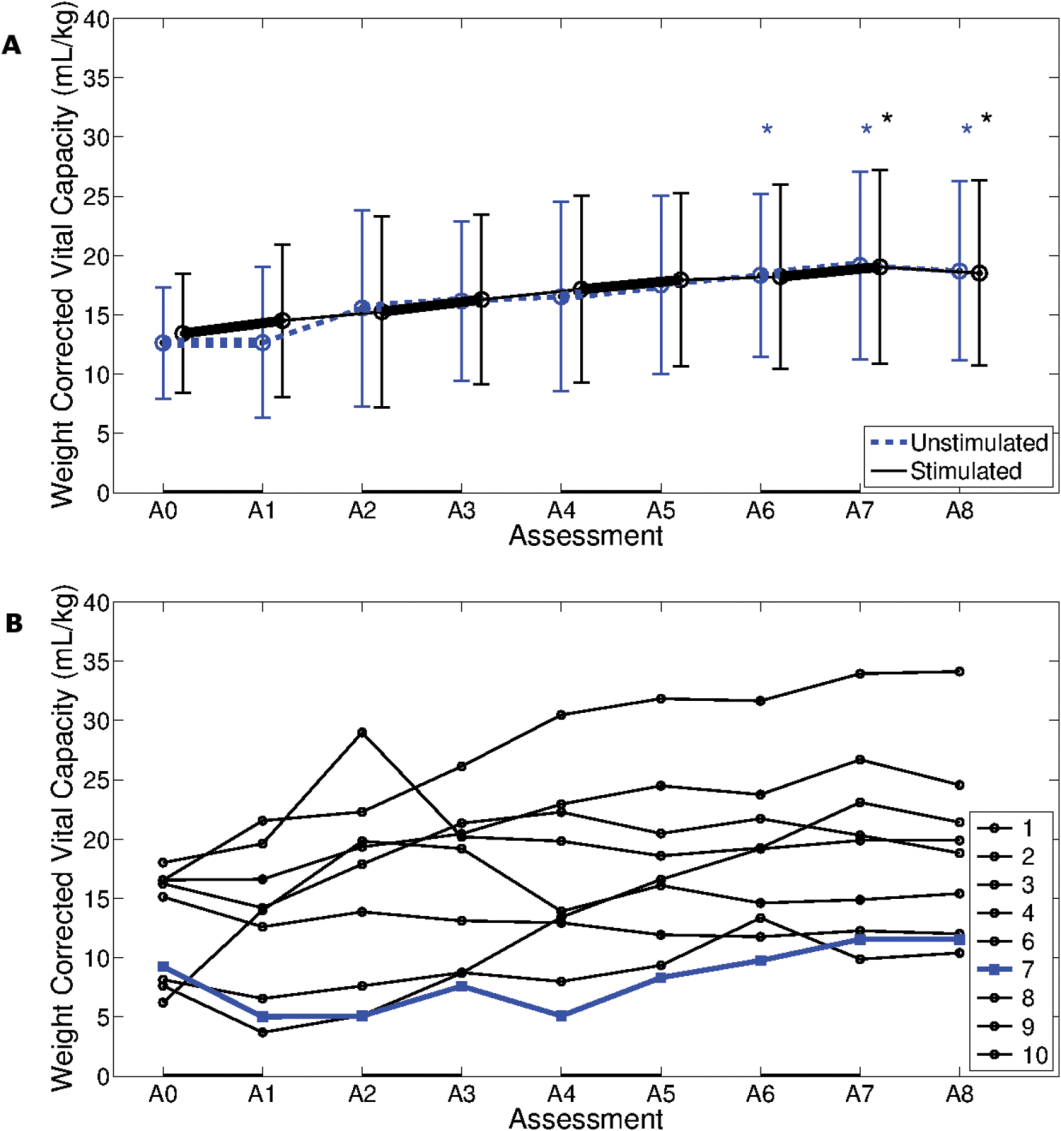
Weight corrected vital capacity. A. Group weight corrected vital capacity (V_*C*_/kg) (mean ± standard deviation) for 9 participants, recorded at 9 weekly assessment sessions. A black line represents stimulated, and a blue line unstimulated, breaths. Black * and blue * indicate that stimulated and unstimulated mean was statistically significantly different to the mean at first assessment, respectively. B. Unstimulated V_*C*_/kg of nine participants, recorded at nine weekly assessment sessions. Participant 7, who was the only participant not to wean from ventilation within the study duration, is represented by a blue line. A solid black line along the bottom of both plots represents one week of AFES training.

Stimulated V_*T*_/kg was not significantly different to unstimulated V_*T*_/kg (*p* = 0.492). Both stimulated and unstimulated V_*C*_/kg increased significantly throughout the study (*p* < 0.005 for both measures), with the stimulated V_*C*_/kg at the final two assessment sessions and the unstimulated V_*C*_/kg at the final three assessment sessions significantly greater than at the first assessment session (see Fig 6A). None of the weekly changes in V_*C*_/kg, or the differences between stimulated and unstimulated V_*C*_/kg at each assessment session, were statistically significant.

Participant 7 showed a consistently low V_*C*_/kg (see Fig 6B).

### Time to wean

The time from initial ventilation for each participant to achieve seven consecutive days breathing without the assistance of mechanical ventilation is shown in Table 2. Intervention participants weaned from mechanical ventilation on average 11 (sd: ± 26) days faster than controls. The difference between the two groups was not statistically significant (*p* = 0.41, Df = 1, Hazard ratio = 0.65). However, this result was regarded as clinically significant.

**Table 2:** Time for each participant to achieve seven days without ventilation from initial date of ventilation.

	Time to wean (days)	
Participant	AFES	Control
1	64	48
2	28	39
3	18	40
4	38	92
6	36	N/A
7	N/A	51
8	31	14
9	27	18
10	23	50
Mean ± s.d	33±14	44±24

Intervention participants who did not wean from ventilation within the 8 week study duration, and controls who did not wean within 100 days post injury, are denoted by N/A. Mean time to wean for each group ± standard deviation, which do not include Participant 7 and control Participant 6, are shown. Abbreviations: s.d, standard deviation.

## Discussion

The aim of this study was to evaluate the clinical feasibility and effectiveness of using an AFES training program with the acute ventilator dependent tetraplegic population to improve respiratory function and assist weaning from mechanical ventilation. The feasibility of this intervention is demonstrated by the application of AFES with nine participants, five times weekly on four alternate weeks, without any negative side effects. The compliance rate of 96.7% is similar to the 97.8% compliance reported by McBain *et al*. [10] when conducting AFES training with a chronic tetraplegic population, demonstrating how well AFES is tolerated by the ventilator dependent tetraplegic population.

### Respiratory function

Stimulated V_*T*_/kg was significantly greater than unstimulated V_*T*_/kg, agreeing with previous studies that also found an acute increase in V_*T*_ during AFES [24,25]. The finding that V_*C*_/kg increased throughout the study also agrees with previous studies that found an increase in V_*C*_ after AFES training [10,11,26].

With any intervention study in the acute spinal cord injured population it is difficult to distinguish between an intervention effect and ‘neurological recovery’. AFES was applied on alternate weeks with the hope this would indicate improvements in respiratory function caused by the intervention, however no significant weekly changes in respiratory function were found. Liaw *et al*. [27] observed a 27% increase in FVC over a six week period for sub-acute tetraplegics who received no intervention, a mean increase of 4.5% per week. In this eight week intervention study it was found that unstimulated V_*C*_/kg increased by 48%, a mean increase of 6% per week. This suggests that there may be an intervention effect from AFES training. While the small sample size in this study means that it is not possible to rule out neurological recovery as a major contributing factor in the longitudinal changes in respiratory function, the increases in respiratory function during weeks where AFES training was not applied may be caused by a combination of neurological recovery, the patient’s physiotherapy and an acute carry over effect from the previous weeks AFES training. Although there is no literature investigating the presence of a carryover effect from AFES, previous studies have reported a carryover effect from FES training of the lower limbs to correct foot drop [28,29].

### Time to wean

Participants who received AFES training weaned from mechanical ventilation on average 11 days faster than matched controls. While the numbers in this study are too small for this difference to reach statistical significance, with limitations in the matching process (see Section Limitations), this indicates that the application of an AFES training program may improve respiratory function to a level that it enables faster weaning from mechanical ventilation. In future studies it may be beneficial to employ a larger sample size and to not match participants retrospectively, which would allow an analysis to be performed on the correlation between time to wean and age, injury level, sex and AIS score.

Participant 7 was the only intervention participant who completed the training protocol and did not wean from mechanical ventilation within the study duration. Interestingly they were the only participant who did not achieve a V_*T*_/kg of greater than 5 mL/kg at any point in the study, suggesting this may be an indicator for weaning success with the tetraplegic population.

### Mechanisms of AFES

It has been suggested that AFES training increases muscle mass, providing greater support to the abdominal contents. As the abdominal contents act as a pivot point for the diaphragm when it contracts, the greater support provided to them by the abdominal muscles places the diaphragm in a more efficient position after contraction [11]. It is also possible that by achieving an acute increase in respiratory function, AFES is assisting motor relearning, teaching the body a more efficient method of respiration. While this study does not provide any additional information regarding the physiological mechanisms of AFES, studies that explore these mechanisms will be vital to further optimise this intervention.

### Other observations

For electrical stimulation to be successful the lower motor neurons must be intact [7]. Participant 5 did not respond to stimulation due to lower motor neuron damage. This participant still requires full time ventilatory support. Participant 7, who did not wean from ventilation during the study, had a large amount of oedema (extra cellular fluid) in the abdomen. As a result, a much larger current than in other participants was required to initiate a contraction of the abdominal muscles (105 mA versus mean of 59 mA for the other participants). This agrees with Harper et al. [30] who showed that, in the presence of oedema, the current required to induce a contraction in the wrist was double that required to initiate a contraction without oedema. This suggests that oedema may reduce the effectiveness of AFES, and that abdominal oedema should be considered a contraindication for the use of AFES. Participant 7 had a cardiac pacemaker fitted, with no negative effects of stimulation observed, agreeing with the findings of Shade [31]. The finding that two out of the 10 intervention participants (20%) did not wean from mechanical ventilation agrees with Gay [3], who reports that every year in the USA 18.5% of new tetraplegics require permanent mechanical ventilation.

Participant 1, who developed pneumonia during week four of participation, was the only participant to develop a respiratory complication during this study. Jackson and Groomes [32] reported that in the first six weeks after injury 175 of 261 (67%) tetraplegic patients developed a respiratory complication. The low numbers of respiratory complications reported here agrees with the finding of Cheng *et al*. [26] who also reported a reduction in respiratory complications after an AFES training program. This suggests that the improvements in respiratory function achieved using an AFES training protocol may prevent respiratory complications.

### Limitations

A major limitation of this study is the low number of participants. Due to the anticipated number of patients who would be eligible for this study in the study time frame it was not possible to form a parallel prospective control group. Participants served as their own controls, with AFES training applied on alternate weeks. This pause in AFES training may have prevented any fibre type conversion, reported to be a beneficial effect of AFES training [7]. As the feasibility of AFES with acute ventilator dependent tetraplegic patients has been demonstrated in this study, a continuous AFES training program should be adopted in future studies, with a parallel control group used in larger clinical trials to replace the application of AFES on alternate weeks.

A retrospective control group was used to assess ventilation duration. While retrospective matching was performed based on injury level, age and sex, due to the relatively small number of retrospective control patients exact matches were difficult to achieve. Clinical judgement was used during the matching process. Additionally, AIS score was not used as a matching criteria. Matching patients with different AIS scores may mean comparing patients who can (AIS C and D) and cannot (AIS A and B) achieve a voluntary abdominal muscle contraction, yet AIS score is not currently reported to be an indicator of ventilation duration in tetraplegia [17–19]. While AIS score was not found to be a predictor of ventilation duration in this study, the authors suggest, through clinical judgement, that AIS score should be considered as a matching criteria for future studies. While the authors acknowledge that larger trials are required to adequately assess the effectiveness of abdominal FES to assist ventilator weaning, they believe that the demonstration of feasibility in this trial is a necessary step towards such trials.

As reliance on mechanical ventilation can cause muscle atrophy, an early intervention should reduce the time spent on mechanical ventilation. This study aimed to recruit participants as soon as possible after ventilation. Participant 10, who started the study two days after ventilation commenced (see Table 1), was the only participant who started in the first five days after ventilation. They were also the second fastest participant to wean from mechanical ventilation in the intervention group, which may indicate the benefits of an early intervention. Many of the participants in this study spent time on a general high dependency ward before being transferred to the spinal unit for specialist care. For Participant 2 and Participant 4 this initial care was provided in another country, resulting in these participants being recruited slightly after the four week acute injury period. Participant 7 was recruited for this study 22 days post injury. However, due to repeated poor health this participant did not begin training until 43 days post injury. In future studies earlier recruitment may lead to a greater benefit from the intervention.

## Conclusions

This study shows for the first time that AFES is a feasible technique for assisting ventilator weaning for people with acute tetraplegia in a clinical setting. AFES training was shown to improve the respiratory function of this population, with a (non-significant) decrease in the time spent dependent on mechanical ventilation compared to historic controls.

## Supporting Information

S1 Protocol Clinical Study Protocol. (PDF)

S1 Statement TREND Statement. (PDF)
