# Supplementary material for: Abdominal Functional Electrical Stimulation to Assist Ventilator Weaning in Acute Tetraplegia: A Cohort Study
Source: PLoS One. 2015 Jun 5;10(6):e0128589. doi: 10.1371/journal.pone.0128589 (PMC4457912; doi:10.1371/journal.pone.0128589)
Supplement: S1 Protocol — (PDF) [file pone.0128589.s001.pdf]

Centre for Rehabilitation Engineering

NHS Greater Glasgow and Clyde

Protocol

Project Title: **Feasibility study using Abdominal Functional Electrical Stimulation (AFES) to assist ventilator weaning in tetraplegia**

Investigators: **Dr. Henrik Gollee [1,2]**

Dr. Alan McLean[2,3], Mr. David Allan [2,3]

Researcher: **Mr. Euan McCaughey [1,2]**

[1] Centre for Rehabilitation Engineering, University of Glasgow, Glasgow, UK

[2] Scottish Centre for Innovation in Spinal Cord Injury, Glasgow, UK

[3] Queen Elizabeth National Spinal Injuries Unit, Southern General Hospital, Glasgow, UK

1 Executive Summary

Compromised respiratory function as a result of tetraplegia leads to many tetraplegics requiring mechanical ventilation during the acute phase of injury. Ventilation costs the local health care provider an additional £1000 per day per patient and reduces a patient's quality of life. Electrical stimulation of the abdominal muscles has previously been used to improve the respiratory function of tetraplegic patients in the chronic stage of injury. In this study we aim to evaluate whether electrical stimulation of the abdominal muscles can assist the process of weaning from mechanical ventilation in acute ventilator dependent spinal cord injured patients.

2 Introduction

Spinal cord injury can lead to paralysis of the limbs and muscles as well as a loss of function and sensation. When a person suffers an injury in the cervical (neck) region of the spinal cord, this is termed tetraplegia. Tetraplegia can lead to paralysis of all four limbs and affect the respiratory function of the patient. Poor respiratory function often leads to a patient with a cervical injury requiring mechanical ventilation and possibly a tracheostomy during the acute phase of injury [1].

Mechanical ventilation costs the local health care provider in the region of an additional £1000 per day for each patient [2]. Mechanical ventilation also reduces the patient's ability to take part in rehabilitation treatment which is a vital step of the recovery process [3]. If the time to wean from the ventilator can be reduced for this patient group this will result in a significant cost saving for the local health care provider, as well as greatly improving the patient's quality of life.

There are currently several methods available to promote weaning from mechanical ventilation. One traditional method to promote weaning is intermittent mandatory ventilation where the patient remains connected to the ventilator but is required to progressively initiate more breaths themselves. Spontaneous breathing trials (SBTs) have been shown to be a successful method to promote faster ventilator weaning than the traditional method of intermittent mandatory ventilation [4-6]. During SBTs the patient is encouraged to breathe on their own several times a day and the duration of this ventilator free time is progressively increased. As the amount of air which the patient can shift is likely to be very small at the initial stages of weaning, only short periods of time without ventilator support can be tolerated. However as the muscles regain strength the time without mechanical ventilation increases until eventually the patient becomes ventilator independent.

Functional Electrical Stimulation (FES) is a method of stimulating the motor nerves of a muscle using a small electrical current in order to gain a functional improvement. Functional electrical stimulation when applied to the abdominal muscles is termed Abdominal Functional Electrical Stimulation (AFES). AFES has been shown to increase tidal volume (amount of air in each breath) in tetraplegia [7,8]. AFES has also been shown to be a suitable approach to improve respiratory function in chronic ventilator dependent tetraplegics without spontaneous ventilation [9]. Recent studies have shown that AFES may be a suitable method to assist ventilator weaning in the ventilator dependent tetraplegic group [10]. By making use of AFES in the acute tetraplegic population, coupled with PVFB, it may be possible to improve tidal volume to such a degree that it reduces the patient's time on a mechanical ventilator.

The total time that each patient spends on mechanical ventilation varies significantly, and there are currently no clearly defined prediction methods for weaning times [11–13]. Some studies predict that injury level, age and sex are the best indicators of the time for which a patient is likely to be ventilator dependent. Therefore a possible approach for predicting weaning time is to match subjects with a retrospective control group based on injury level, age and sex.

In summary the use of AFES in acute ventilator dependent spinal cord injured patients could support faster weaning from mechanical ventilation, with the potential to reduce health care costs and significantly improve the patient's quality of life.

3 Aims

The two main aims of this study are to evaluate the feasibility and effectiveness of abdominal Functional Electrical Stimulation to assist faster weaning from mechanical ventilation in acute ventilator dependent tetraplegic subjects.

Secondary aims of this study are (i) the evaluation of the engineering methods which have been developed to allow the synchronization of abdominal functional stimulation with a mechanical ventilator, and (ii) to investigate whether regular applications of abdominal functional electrical stimulation can improve the respiratory function of acute ventilator dependent tetraplegic patients.

4 Methods

4.1 Participants

10 ventilator dependent tetraplegic participants in the acute stage of injury will be recruited while inpatients at the Queen Elizabeth National Spinal Injuries Unit (QENSIU), Southern General Hospital, Glasgow. The participants will then be matched for injury level, age (+/-5 years) and sex against a control obtained from records of previous patients in the QENSIU. The control data will be collected by Dr Alan McLean and the researchers will not have access to patient notes at any point during the study.

The participants will be recruited according to the following inclusion and exclusion criteria.

Inclusion criteria:

- Men or women over 16 years of age;
- Acute spinal cord injury (Patients with a new spinal cord injury);
- Reduced respiratory function requiring mechanical ventilation;
- Good visual response to surface abdominal stimulation, suggesting that lower motor neurons are intact.

Exclusion criteria:

- Under 16 years of age;
- Female subjects who are pregnant;
- Significant history of autonomic dysreflexia;
- No visual response to surface abdominal stimulation, suggesting that lower motor neurons are not intact.

4.2 Experimental Procedures

Each participant will take part in daily AFES training sessions (5 times per week) during alternative weeks. Progress will be monitored during weekly assessment sessions. The total duration of participation is 8 weeks.

4.2.1 Weaning sessions

The aim of the weaning sessions is to strengthen the abdominal muscles and to provide support for ventilator free breathing.

The participant will receive AFES training once per day for a pre-determined time (see below), 4 days per week (Monday to Thursday, with rest days on Saturday and Sunday). Before the study begins and at the end of each week an assessment session (see below) will be conducted on a Friday. The participant will then spend 1 week without receiving AFES training. This cycle of 1 week with AFES training and 1 week without will give an indication of the effectiveness of AFES on the weaning progress. The total time of participation will be 8 weeks as shown in Figure 1.

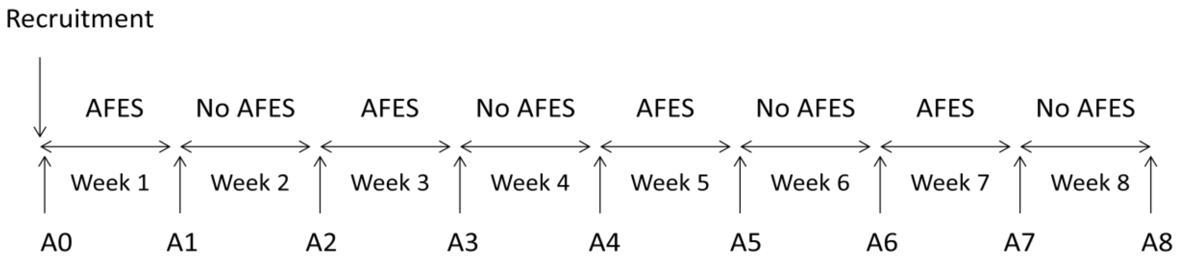

Figure 1: Study timeline

AFES training sessions will continue throughout even if the patient is successfully weaned before the end of the 8 week period. All procedures will be carried out at the participant's bedside and during all procedures a clinician and the participant's nurse will be present.

The duration of AFES training will initially be 20 minutes. This will be increased to 30 minutes in week 3 and to 40 minutes during weeks 5 and 7.

During the study the participant will undergo SBTs during the weaning sessions. During these trials the participant is likely to go through four distinct phases before being successfully weaned from the ventilator as outlined in Table 1 below (adapted from [4-6]).

1. Participant unable to breathe independently of ventilator but with gradually decreasing ventilator support	Participant will not be able to breathe independently of the ventilator. Therefore all procedures will be carried out with the participant connected to the ventilator.
2. Participant taken off ventilator for the first time and SBTs begin	Participant will only be able to breathe independently of the ventilator for a short period of time before SaPO2 falls below 92%. It is unlikely that the participant will be able to actively participate or follow instructions. All breaths will be at vital capacity.
3. Participant becomes more accustomed to breathing without ventilator support for longer periods of time	Duration of time off the ventilator increases before SaPO2 falls to 92%. However breaths are still at vital capacity until respiratory function begins to improve.
4. Participant can actively participant while off ventilator	Participant can spend the whole session disconnected from the ventilator. Respiratory

	function improves to level where tidal volume of normal breath is below vital capacity. Participant can actively take deeper breaths to vital capacity when requested.

Table 1: Phases of weaning.

While the participant is still not able to breathe independently of the ventilator, Phase 1 of Table 1, AFES will be applied while the participant is connected to the ventilator. However throughout Phase 1 the ventilator support will be reduced and the weaning process will begin. As weaning progresses, Phase 2,3 and 4 of Table 1, SBTs will begin and AFES will be applied with the participant disconnected from the ventilator. While the participant is off the ventilator their oxygen saturation level ($SaPO_2$) will be monitored and recorded every minute. If the participant's $SaPO_2$ level falls below 92% the participant will be immediately reconnected to the ventilator until the $SaPO_2$ returns to the initial value. A previous study by Girard et al. [6] suggested a SaPO2 of 88% as the point at which heterogeneous participants in an ICU should be reventilated during SBTs. However inline with current clinical procedures at the QENSIU, SaPO2 of 92% was deemed a more appropriate level at which to reventilate the participant during this study.

The participant's weaning progress will be monitored throughout the course of the study. Three measures of ventilator weaning, adapted from a previous study by Narh et al. [11], will be used to determine whether weaning has been successful: The participant is (i) *awake weaned* when the they are able to breathe independently of the ventilator during the day, (ii) *short term weaned* when they do not require ventilator for 24 hours, and (ii) *long term weaned* when they have been ventilator free for 7 days. Based on previous experience most participants will not need to be reventilated after short term weaning has been achieved, although this may be compromised by comorbidities such as respiratory infection. Weaning progress is recorded in the patient notes, allowing a comparison to be made between the participants and the historic control group.

4.2.2 Assessment sessions

An initial assessment session, represented by A0 on Figure 1, will be conducted on day 1 of the study to provide a baseline measure of the participant's respiratory function. Assessment sessions will then be conducted once per week to assess weaning progress as shown in Figure 1. The assessment sessions will be carried out in place of a regular weaning session, and may be augmented with a period of AFES training to ensure that the prescribed period of AFES is achieved (see above).

During the assessment sessions two breathing modes will be assessed.

1. *Unassisted breathing*: The participant will be asked to breathe without ventilator support. Tidal volume (amount of air inhaled and exhaled during a breath, V_T) and vital capacity (total lung capacity, V_C) will be measured. It is expected that while the participant is in Phase1, 2 and 3 of weaning (Table 1), V_T and V_C will be similar. Once the participant moves onto Phase 4 the V_C will become larger than V_T as the participant begins to be able to breathe more efficiently. V_T, together with breathing rate, will be measured during normal ventilator free breathing. During Phase 4, V_C will be assessed by asking the participant to inhale to maximum volume and then fully exhale.

2. *AFES assisted breathing*: The participant will be asked to breathe on their own without the support of the ventilator, but with the assistance of abdominal FES. The same measurements as for unassisted breathing will be recorded.

Each of the breathing modes will be assessed for 6 minutes or until the participant's $SaPO_2$ level drops below 92%. Both modes will be separated by a break of at least 2 minutes duration, or until the participants $SaPO_2$ returns to the starting level. Participants will be ventilated during this break if required.

4.3 Apparatus

A programmable neuromuscular stimulator (Rehastim, Hasomed, Germany) will be used during the weaning sessions. Stimulation intensity will be adjusted on a channel by channel basis until a strong visible contraction of the abdominal muscles is observed. The stimulator will be synchronised with the mechanical ventilator using a custom interface (based on a pressure sensor to distinguish inspiratory and expiratory periods). During the session, the program allows adjustment of both the stimulation intensity (to compensate for fatigue) and the duty cycle. The stimulator is CE marked, being used for its intended use, and is regularly serviced by the manufacturer. The stimulator has also been approved for use in a previous study by the medical physics department of the Southern General Hospital, Glasgow. The pressure sensor is also CE marked and being used for its intended use.

For the weaning and assessment sessions four pairs of surface electrodes (PALS, Axelgaard) will be placed on the participant's abdomen. One pair will be placed on both the right and left side of the rectus abdominis around the naval with a separation between each electrode of around 3cm. One pair of electrodes will also be placed on both the right and left side of the external oblique muscles with one electrode being placed just below the participant's bottom rib and the other below this with a separation of around 3cm. Each patient on the study will have their own set of electrodes. The electrodes are CE marked and are being used for their intended use. The electrodes have also been approved for use in a previous study by the medical physics department of the Southern General Hospital, Glasgow.

In the initial assessment sessions a spirometer (Microloop, Micromedical,Chatham,UK) will be used to measure the participant’s respiratory flow and tidal volume for a few breaths without mechanical ventilation. Depending on the ventilation mode, the spirometer will be connected either to a low dead-space full face mask worn by the participant, or (if present) to the participant's tracheostomy tube. If the tracheostomy is uncuffed it may be necessary for the participant to wear a noise clip to avoid any air leakage. The measurements will be recorded on a laptop. The stimulator will also be connected to the laptop and controlled by custom software, allowing adjustments to timing and intensity. The spirometer is CE marked, being used for its intended use, and is regularly serviced by the manufacturer. The spirometer has also been approved for use in a previous study by the medical physics department of the Southern General Hospital, Glasgow.

4.4 Outcome measures

The main outcome measure of this investigation will be the time taken to wean from mechanical ventilation. Secondary outcome variables will be measures of respiratory function recorded throughout the intervention period.

4.5 Statistical considerations

Standard sample size formulae do not apply for this feasibility study design. The variability of time to wean from ventilation in the tetraplegic population and the fact that the quantitative effectiveness of the intervention is largely unknown in this patient group make it difficult to reliably estimate the required sample size. The target number of patients is therefore mainly determined by an estimate of the available patient population at QENSIU whom may be eligible for this study.

5 Ethical aspects

5.1 Consent

All potential participants in this study will be under the care of a spinal cord injury consultant. This consultant will identify potential participants and will inform Mr Allan (QENSIU Director), who will confirm a participant's eligibility for the study. Participants will remain under the care of their spinal cord injury consultant but medical care during experimental procedures will be under the supervision of Dr Alan McLean (Spinal cord injury consultant with an interest in respiratory care).

Due to the effect of mechanical ventilation all potential participants will be incapable of giving verbal consent and will be physically incapacitated. All participants on the study will be certified (Certificate of Incapacity under Section 47 of the Adults with Incapacity (Scotland) Act 2000) as being incapacitated in conformity to the 'Adults with Incapacity (Scotland) Act 2000' by a spinal cord injuries consultant unrelated to the study. The participant's consultant will inform the participant's welfare guardian, or in the case no welfare guardian is appointed, nearest relative, of the participant's eligibility for the study and give the welfare guardian/nearest relative an overview of the study design.

In accordance with Section 51 of the Adults with Incapacity (Scotland) Act 2000 the participant's welfare guardian will be responsible for giving consent for the participant to take part in the study. In the case where no welfare guardian exists a nearest relative may give consent in the condition no nearer relative or welfare guardian exists.

If the welfare guardian/ nearest relative indicates to the participant's consultant that they would be willing to allow the participant to take part in the study the researchers will be introduced to the potential participant and their welfare guardian/nearest relative by the participant's consultant. The researchers will meet the welfare guardian/nearest relative and will provide them with an information sheet and invitation letter and discuss the intervention. The welfare guardian/nearest relative will then be asked to sign a welfare guardian/nearest relative consent form on behalf of the participant.

The welfare guardian/nearest relative of the participant may withdraw their consent for the participant to be part of the study at any time. Throughout the duration of the study any family member may approach the researchers at any point to discuss the intervention.

Participant's capacity will be reassessed on a daily basis by a spinal cord injury consultant during their ward round. When the participant is deemed as having regained capacity the researchers will meet the participant again. The participant will be provided with an information sheet and a letter informing them of why they were taking part in the study. The

participant will then be free to ask the researchers any questions relating to the study. The participant will then be invited to sign a reconsent form if they wish to continue participating in the study. If the participant decides they no longer wish to take part in the study they will be given the option of having the data collected thus far kept or destroyed. After regaining capacity and signing the reconsent form the participant will be free to withdraw from the study at any point, without reason.

Photographs of the participant may be taken and used in publications, presentations or on websites where this research will be disseminated. The participant's welfare guardian/nearest relative will indicate their consent for photographs to be taken in the welfare guardian/nearest relative consent form. Photographs will only be taken of the equipment set up and in any photograph the participant will not be able to be identified.

Although initially participants on the study will lack the ability to consent it is anticipated that the success of the intervention will speed up a patient's recovery, which in turn will reduce the time that they are certified as incapacitated and allow earlier consent to be given. Therefore this research is in accordance with Section 51 of the Adults with Incapacity (Scotland) Act 2000.

5.2 Procedures

The risks of this study are deemed to be low. Participants will be given abdominal FES at least 5 times per week, on alternate weeks, over an eight week period. These will be arranged to minimise any interference with their regular care and rehabilitation and will be carried out at the participant's bedside.

Electrical muscle stimulation using surface electrodes is routinely used for the stimulation of muscle in people with an SCI. Provided safety guidelines are followed it is not known to be harmful or present a risk to the participant. It will generally not cause discomfort in complete tetraplegia, since the participants will lack sensation at the stimulation site. If the participant is sensitive to the stimulation, the intensity will be kept at a level which is comfortable.

Any procedure in tetraplegia carries a risk of causing autonomic dysreflexia (AD), a form of hypertension which can be triggered by any kind of stimulation. The risk of an AD event will be minimised by the application of the inclusion/exclusion criteria. All investigators will be aware of the potential for dysreflexia and treatment required, and the required medication will be available during the procedures.

During any period which the participant spends without the aid of mechanical ventilation it is possible that there will be a significant fall in the oxygen saturation level ($SaPO_2$). $SaPO_2$ levels will be monitored throughout the tests and recorded every minute. If the participant's $SaPO_2$ level drops below 92% the participant will be immediately reconnected to the ventilator until the $SaPO_2$ returns to the initial value.

5.3 Emergency procedures

The overall risk of harm to participants in this study is deemed to be low. All procedures will be carried out at the participant's bedside on the ward. A clinician and the participant's nurse will be present during all procedures. However, in the event of an untoward incident arising

that is deemed an emergency, testing will be stopped and the Southern General Hospital Emergency Response Team will be summoned by telephone. In the event of an untoward incident arising that is not deemed an emergency, testing will be stopped and a member of the Queen Elizabeth National Spinal Injuries Unit on call team will be summoned to assist the clinician in dealing with this problem. All the researchers involved in this study have attended an induction course designed for SHO's and are fully aware of the emergency procedure.

5.4 Data protection

Data relating to participants will be anonymised using a study ID and used only within the research study. Data will be stored in password-protected computers. Computers will also be stored in a locked office. All consent forms and experimental notes will be stored within a locked filing cabinet in the research mezzanine at the QENSIU. The chief investigator will act as custodian of the data.

6 References

[1] Rainer O Seidl, DianaWolf, Ricki Nusser-Mller-Busch, and Andreas Niedeggen. Airway management in acute tetraplegics: a retrospective study. Eur Spine J, 19(7):1073–1078, Jul 2010.

[2] Joseph F Dasta, Trent P McLaughlin, Samir H Mody, and Catherine Tak Piech. Daily cost of an intensive care unit day: the contribution of mechanical ventilation. Crit Care Med, 33(6):1266– 1271, Jun 2005.

[3] S. Berney, P. Bragge, C. Granger, H. Opdam, and L. Denehy. The acute respiratory management of cervical spinal cord injury in the first 6 weeks after injury: a systematic review. Spinal Cord, 49(1):17–29, Jan 2011.

[4] A.Esteban et al. A comparison of four methods of weaning patients from mechanical ventilation. N Engl J Med, 332, 345-350, 1995.

[5] M.O. Meade, G.H. Guyatt & D.J. Cook, Weaning from mechanical ventilation: the evidence from clinical research. *Respir Care, 46*, 1408-15, 2001.

[6] T.D Girard & E.W. Ely. Protocol-driven ventilator weaning: reviewing the evidence. *Clin Chest Med, 29*, 241-52, 2008.

[7] Pao-Tsai Cheng, Chia-Ling Chen, Chin-Man Wang, and Chia-Ying Chung. Effect of neuromuscular electrical stimulation on cough capacity and pulmonary function in patients with acute cervical cord injury. J. Rehab. Med., 38(1):32–36, 2006.

[8] H. Gollee, K. J. Hunt, D. B. Allan, M. H. Fraser, and A. N. McLean. Automatic electrical stimulation of abdominal wall muscles increases tidal volume and cough peak flow in tetraplegia. Technol Health Care, 16(4):273–281, 2008.

[9] F. Kandare, G. Exner, J. Jeraj, A. Aliverti,R. Dellaca, U. Stanič, A. Pedotti, & R. Jaeger. Breathing Induced by Abdominal Muscle Stimulation in Individuals Without Spontaneous Ventilation. Neuromodulation, *5*, 180-185, 2002.

[10] Bonsan B Lee, Claire Boswell-Ruys, Jane E Butler, and Simon C Gandevia. Surface functional electrical stimulation of the abdominal muscles to enhance cough and assist tracheostomy decannulation after high-level spinal cord injury. J Spinal Cord Med, 31(1):78–82, 2008.

[11] E Atito-Narh, S Pieri-Davies, and JWH Watt. Slow ventilator weaning after cervical spinal cord injury. British Journal of Intensive Care, Autumn 2008:95–102, 2008.

[12] Anthony E. Chiodo, William Scelza, and Martin Forchheimer. Predictors of ventilator weaning in individuals with high cervical spinal cord injury. Journal of Spinal Cord Medicine, 31:72–77, 2008.

[13] WP Peterson, L Barbalata, CA Brooks, KA Gerhart, DC Mellick, and GG Whiteneck. The effect of tidal volumes on the time to wean persons with high tetraplegia from ventilators. Spinal Cord, 37:284–288, 1999.
